# Susceptibility to an inoculum of infectious hypodermal and haematopoietic necrosis virus (IHHNV) in three batches of whiteleg shrimp *Litopenaeus vannamei* (Boone, 1931)

**DOI:** 10.3897/zookeys.457.6715

**Published:** 2014-11-25

**Authors:** César Marcial Escobedo-Bonilla, José Luis Ibarra Rangel

**Affiliations:** 1 Blvd. Juan de Dios Bátiz Paredes número 250, Colonia San Joachin. Código Postal 81101, Guasave Sinaloa, México

**Keywords:** *Litopenaeus vannamei*, shrimp batches, shrimp susceptibility, IHHNV, infectivity titer, pathology

## Abstract

The present study evaluated the susceptibility of three different batches of whiteleg shrimp *Litopenaeus vannamei* from Mexico to an inoculum of infectious hypodermal and haematopoietic necrosis virus (IHHNV). Each of the three shrimp batches came from a different hatchery. Because of their origin, it was possible that the genetic makeup of these batches was different among each other. The three batches tested showed differences in IHHNV susceptibility. Here, susceptibility is defined as the capacity of the host to become infected, and it can be measured by the infectivity titer. Susceptibility to IHHNV was observed in decreasing order in shrimp from batch 1 (hatchery from El Rosario, Sinaloa), batch 3 (hatchery from Nayarit) and batch 2 (hatchery from El Walamo, Sinaloa), respectively. The largest susceptibility difference between batches was 5012 times, and that between early and late juveniles from the same batch was 25 times. These results indicate that within a species, susceptibility to a pathogen such as IHHNV can have large differences. Susceptibility to pathogens is an important trait to consider before performing studies on pathogenesis. It may influence virological parameters such as speed of replication, pathogenicity and virus titer. In order to evaluate the potential use of IHHNV as a natural control agent against white spot syndrome virus (WSSV), it is necessary to know host susceptibility and the kinetics of IHHNV infection. These features can help to determine the conditions in which IHHNV could be used as antagonist in a WSSV infection.

## Introduction

Infectious hypodermal and haematopoietic necrosis virus (IHHNV) was first detected in stocks of the blue shrimp *Litopenaeus stylirostris* in Hawaii in 1980, where it caused high mortalities (Lightner et al. 1983). Later, it was observed that IHHNV induced runt deformity syndrome (RDS) in infected whiteleg shrimp *Litopenaeus vannamei* (see Kalagayan et al. 1991). In Mexico, mortalities caused by IHHNV were so severe that farmers were forced to switch from the widely cultured blue shrimp to the less cultured whiteleg shrimp. The latter species soon became the main cultured species since the late 1980s (Lotz 1997). This virus has affected other penaeid species in the Pacific (Alcivar-Warren et al. 1997) and various countries in America, Asia and Oceania (Lightner 1996).

Records from the Gulf of California indicate that IHHNV appeared later than 1987 (see Morales-Covarrubias et al. 1999a). In 1990, the virus was present in hatcheries and farms from Sinaloa and Sonora, where the two shrimp species (whiteleg or blue) were grown. The same year, IHHNV prevalence in wild populations of *Litopenaeus vannamei*, *Litopenaeus stylirostris* and *Farfantepenaeus californiensis* from the Gulf of California varied from 46% to 26% (Pantoja et al. 1999). In 1996, IHHNV prevalence in the Platanitos area in Nayarit was up to 86–89% in wild adult *Litopenaeus stylirostris* females (Morales-Covarrubias et al. 1999b). In the year 2000, a survey carried out off the coast of Panama in wild *Litopenaeus vannamei* broodstock showed that IHHNV prevalence was 28% (Nunan et al. 2001). Other locations where IHHNV has been reported include Colombia (1990), Texas (1991), Ecuador (1992), Panama (1997) (see Tang and Lightner 2002), Australia (1992, 1993) (see Owens et al. 1992, Krabsetsve et al. 2004); Philippines (1996), Thailand (2000), Taiwan (2001), Tanzania (2000), Madagascar (2000), Mauritius (2000) (see Tang et al. 2003a), Argentina (2003–2007) (see Martorelli et al. 2010) and China (2007) (see Yang et al. 2007). Shrimp species reported to be affected by IHHNV are: farmed and wild specimens of *Litopenaeus vannamei*, *Litopenaeus stylirostris*, *Penaeus monodon*, *Penaeus semisulcatus*,﻿ wild *Artemesia longinaris*, *Farfantepenaeus californiensis*, and hybrid *Penaeus monodon* × *Penaeus esculentus* (Escobedo-Bonilla 2013).

The IHHNV belongs to the family Parvoviridae (Bonami et al. 1990). It has an icosahedral shape, 22 nm in diameter with a single-stranded DNA molecule. Its genome is 4075 nucleotides long (Shike et al. 2000, Tang and Lightner 2006, Robles-Sikisaka et al. 2010), which makes it one of the smallest genomes known for viruses. Since the spread of this virus to other countries, its prevalence in farmed populations of *Litopenaeus vannamei* has remained steady without obvious clinical signs of disease (Kalagayan et al. 1991).

Currently, IHHNV does not cause high mortalities in farmed populations of blue (*Litopenaeus stylirostris*) or white (*Litopenaeus vannamei*) shrimp. Conversely, recent reports indicate that IHHNV infection may protect shrimp against a subsequent white spot syndrome virus (WSSV) infection reducing mortality (Tang et al. 2003b, Bonnichon et al. 2006, Melena et al. 2006). At present, WSSV is the most lethal pathogen to cultured shrimp since its discovery in 1992 (Escobedo-Bonilla et al. 2008, Lightner 2011).

Despite the fact that IHHNV has been present in shrimp farming for over 30 years, little is known about certain features, such as shrimp susceptibility to the virus and speed of virus replication. Here, susceptibility is defined as the capacity of the host to become infected, and it can be measured by the infectivity titer (White and Fenner 1994). It is important to know these IHHNV traits in order to determine whether this virus could be used as antagonist in a WSSV infection using known infectious doses.

Studies carried out with IHHNV compared shrimp susceptibility between species such as the *Litopenaeus vannamei* and the black tiger shrimp *Penaeus monodon* in Thailand. Methods used to determine IHHNV infection were histology, *in situ* hybridization and transmission electronic microscopy. Chayaburakul et al. (2005) found that *Penaeus monodon* was far less susceptible to IHHNV than the whiteleg shrimp. Likewise, IHHNV susceptibility in the Brazilian species *Farfantepenaeus subtilis* was determined through PCR and bioassays (Coelho et al. 2009). In this species, IHHNV infection was transient for 10 d post inoculation (dpi) when low mortalities were reported (7.5–10%), and a few shrimp (3–7.5%) were IHHNV-positive. After 10 dpi, no IHHNV-infected animals were detected by PCR and no mortalities were recorded (Coelho et al. 2009).

Susceptibility differences to IHHNV and *Baculovirus penaei* were assessed within families of *Litopenaeus vannamei* (see Alcivar-Warren et al. 1997): crosses of five families of high-health shrimp with high growth rate (family 1.3) or low growth rate (family 1.6) were evaluated. This study found a relationship between levels of genetic diversity determined by restriction analysis polymorphism and IHHNV prevalence. The highest prevalence of IHHNV occurred in the crossing with lower genetic diversity. In contrast, the lowest IHHNV prevalence was observed in crossing with highest genetic diversity (Alcivar-Warren et al. 1997).

The present study aimed to determine under experimental conditions the susceptibility to an IHHNV inoculum in Mexican batches of whiteleg shrimp *Litopenaeus vannamei* from three different hatcheries.

## Methods

### Shrimp batches

Three batches of Mexican *Litopenaeus vannamei* were used to evaluate their susceptibility to IHHNV. Information about these batches, including mean weight, is presented in Table 1. Each batch was transported to the Laboratory of Aquaculture, CIIDIR Unidad Sinaloa where shrimp were placed in 1000 l tanks provided with a recirculation system. Shrimp were acclimated in seawater at a salinity of 25 g/l, temperature ≤ 30 °C and constant areation. Upon arrival to the laboratory, 30 shrimp from each batch were individually screened through PCR analyses to determine its sanitary status. All batches were negative to IHHNV and WSSV.

**Table 1.** Hatcheries and origin of the three *Litopenaeus vannamei* batches used in the experiments.

Batch	Origin	Collection site	Mean body weight at collection (g)
1	El Rosario, Sinaloa	Granja Aracelitas, Guasave	1.4
2	El Walamo, Sinaloa	El Walamo, Sinaloa	0.7
3	Nayarit, Mexico	Acuícola Machado, Guasave	2.3

### Shrimp maintenance and experimental conditions

Animals were fed at a rate of 5% biomass with pelleted feed (Camaronina 30% Purina, Mexico), split into two daily rations (9:00 h in the morning and 17:00 h in the evening). Tanks had a recirculation system, mechanic filtration and continuous areation. Shrimp were maintained under these conditions until used in the susceptibility assays. Experimental conditions during IHHNV challenge assays were: temperature 27 ± 2 °C, salinity 25 g/l and constant areation. Water exchange (70%) was done every week. Shrimp maintenance was assured by feeding each shrimp with three pellets in the morning and three pellets in the evening.

### Virus inoculum

An IHHNV inoculum was produced from naturally infected shrimp collected from a farm located in Guasave, Mexico in 2010. The inoculum was produced according to the methods described by Escobedo-Bonilla et al. (2005). The IHHNV-infected shrimp as determined by PCR were used. Tissues from the peraeon without cuticle and hepatopancreas were minced and homogenized in nine volumes of 2X phosphate buffered saline (PBS) (174 mM NaCl, 5.4 mM KCl 2.7, 20 mM Na_2_HPO_4_, 4 mM KH_2_PO_4_, pH 7.4). The suspension was clarified by centrifugation (Labnet PrismR, NJ, USA) at 4,000 × g for 10 min at 4 °C and 13,000 × g for 20 min. at 4 °C and filtered through 0.45 µm. The suspension was aliquoted in 1.8 ml cryotubes and stored at -70 °C until used.

### In vivo titration

*In vivo* titration was performed according to the methods described by Escobedo-Bonilla et al. (2005). Five serial dilutions (10^-1^–10^-5^) of the IHHNV inoculum were prepared with PBS. Per dilution, five shrimp were individually kept in 19 l plastic tanks containing 12 l seawater (salinity 25 g/l, temperature 27 ± 2 °C). Each dilution was intramuscularly inoculated (100 µl) to the respective shrimp. Animals were followed for 20 d to determine the time at which they became infected. To do this, a pleopod was collected at 5, 10, 15 and 20 dpi. Total DNA was extracted and used for PCR analyses. Infection data were used to determine the shrimp infectious titer 50% endpoint (SID_50_/ml) according to the Reed and Muench method (Reed and Muench 1938).

### DNA extraction

DNA extraction was carried out with pleopod tissues using DNAzol (MRC Cincinnati, OH, USA) following manufacturer’s instructions. Individual pleopods were homogenized in 1.5 ml Eppendorf tubes containing 500 µl DNAzol, incubated 10 min at room temperature, centrifuged at 13,000 × g for 10 min. DNA was precipitated with absolute ethanol mixing by inversion and incubating at room temperature for 3 min and centrifuged at 7,500 × g for 5 min. The pellet was washed twice with 75% ethanol and centrifuged at 13,000 × g for 2 min. The pellet was air-dried and resuspended in 30 µl ultrapure water (Life Technologies, USA).

### PCR analyses

The IHHNV and/or WSSV PCR analyses were done in 200 µl microtubes. Each microtube contained 24 µl of the PCR mix [18.8 µl ultrapure water, 2.5 µl 10X PCR buffer (Biolase, Irvine CA, USA), 1.0 µl MgCl_2_ (50 mM Biolase, USA), 0.5 µl dNTPs (10 mM, Biolase, USA), 0.5 µl of each primer: IHHNV392F (5’-gggCgAACCAgAATCACTTA-3’), IHHNV392R (5’ ATCCggAggAATCTgATgTg 3’) (Tang et al. 2000), or in-house designed WSSV *vp28* gene primer pair: VP28F1 (5’ CTCTTTCggTCgTgTCggCC 3’) and VP28R2 (5’gAgACgggggTgAAggAggAgg 3’) (Escobedo-Bonilla, unpubl.), and 0.2 µl Taq DNA polymerase]. Each tube contained 1.0 µl DNA from each of the samples as temfig. Total reaction volume was 25 µl. For both viruses, PCR was performed under the following conditions: initial denaturation 95 °C for 4 min. Then, 35 cycles with the following: denaturation 95 °C for 30 s, annealing at 55 °C for 30 s, extension at 72 °C for 45 s and a final extension step at 72 °C for 5 min. The reaction was stopped at 4 °C. The PCR product for IHHNV was a band of size of 392 bp, whereas the PCR product for WSSV was 300 bp.

### Evaluation of IHHNV susceptibility

PCR infection data was used to determine the infectivity titer (SID_50_/ml) for each shrimp batch. Susceptibility of shrimp batches to IHHNV was determined with their respective infectivity titers in the different shrimp sizes.

## Results

By intramuscular inoculation, 15 d were required to determine IHHNV infectivity titer in susceptible shrimp. Juvenile shrimp (≤ 4.0 g) from Batch 1 had an infectivity titer of 10^5.2^ SID_50_/ml, whereas late juveniles (≥ 8.0 g) had an infectivity titer of 10^4.6^ SID_50_/ml (Table 2). In Batch 2, IHHNV infectivity titer was < 10^1.5^ SID_50_/ml since none of the early (≤ 4 g) or late (≥ 8 g) juvenile shrimp inoculated with IHHNV dilutions became infected (Table 3). Shrimp from Batch 3 had an infectivity titer of 10^3.6^ SID_50_/ml in early juveniles (≤ 4g) and 10^2.2^ SID_50_/ml in late juveniles (≥ 8.0 g) (Table 4). Highest susceptibility to IHHNV infection was recorded in Batch 1, followed by Batch 3, and Batch 2 with the lowest susceptibility (Table 5).

**Table 2.** The IHHNV infectivity titers in *Litopenaeus vannamei* Batch 1.

Mean Weight (g)	Dilution	Inoculated shrimp	Infected shrimp	Infectivity titer
3.2 ± 0.78	10^-1^	5	5	10^5.2^ SID_50_/ml
	10^-2^	5	5	
	10^-3^	5	4	
	10^-4^	5	4	
	10^-5^	5	0	
10.5 ± 0.85	10^-1^	5	5	10^4.6^ SID_50_/ml
	10^-2^	4	4	
	10^-3^	5	4	
	10^-4^	5	0	
	10^-5^	5	0	

**Table 3.** The IHHNV infectivity titers in *Litopenaeus vannamei* Batch 2.

Mean Weight (g)	Dilution	Inoculated shrimp	Infected shrimp	Infectivity titer
	10^0^	5	5	
2.9 ± 0.48	10^-1^	5	0	< 10^1.5^ SID_50_/ml
	10^-2^	5	0	
	10^-3^	5	0	
	10^-4^	5	0	
	10^-5^	5	0	
	10^0^	5	5	
8.9 ± 0.76	10^-1^	5	0	< 10^1.5^ SID_50_/ml
	10^-2^	4	0	
	10^-3^	5	0	
	10^-4^	5	0	
	10^-5^	5	0	

**Table 4.** The IHHNV infectivity titers in *Litopenaeus vannamei* Batch 3.

Size (g)	Dilution	Inoculated shrimp	Infected shrimp	Infectivity titer
	10^-1^	5	5	
	10^-2^	5	5	
3.1 ± 0.59 g	10^-3^	5	1	10^3.6^ SID_50_/ml
	10^-4^	5	0	
	10^-5^	5	0	
	10^-1^	5	3	
	10^-2^	5	0	
	10^-3^	5	0	10^2.2^ SID_50_/ml
7.9 ± 0.33 g	10^-4^	5	0	
	10^-5^	5	0	

**Table 5.** Susceptibility differences to IHHNV in *Litopenaeus vannamei* batches.

Batch combination	Same batch early vs. late juveniles	Between batches early juveniles	Between batches late juveniles
1	4 times		
2	----		
3	25 times		
1 vs. 2		5,012 times	1,259 times
1 vs. 3		40 times	251 times
2 vs. 3		126 times	5 times

## Discussion

The three batches used in the present study came from different brooder stocks located in hatcheries that were at least one-hundred kilometers away from each other. Moreover, most hatcheries have their own brooder stock programs, where they avoid using shrimp from other hatcheries. Therefore, it can be assumed that their genetic makeup is different from each other. The three shrimp batches were maintained under the same controlled environmental conditions (temperature, salinity and areation) and the same IHHNV isolate was used. Therefore, shrimp origin is the variable that explains the susceptibility differences found in this study.

Susceptible shrimp showed higher IHHNV susceptibility at early stages within a single batch (≤ 4.0 g mean body weight) (Tables 2, 4). The relationship between shrimp size and virus susceptibility was first observed in batches of whiteleg shrimp in Hawaii (Kalagayan et al. 1991). A similar finding was also described in blue shrimp: animals of 1 to 5 g were more likely to die due to an IHHNV infection than 14 g juvenile shrimp (Bell and Lightner 1987).

Our results revealed that important intraspecific differences in virus susceptibility might occur. Studying host susceptibility is important since it may influence virological traits such as speed of virus replication, pathogenicity and virulence (White and Fenner 1994). These features determine viral pathogenesis and the outcome of infection. A species with variable susceptibility may produce different responses to a pathogen. Such responses can provide new information on virus pathology and pathogenesis.

A number of factors were mentioned to determine differences in host susceptibility to a pathogen: genetic (presence of defense genes, genetic diversity) (White and Fenner 1994, Alcivar-Warren et al. 1997), physiology (age, size, nutrition, stress) (Lotz 1997), environment (water quality) and pathogen persistence (Flegel 2007). In the present study, shrimp was the only variable in our susceptibility experiments, since virus isolate and environmental factors were the same. Although the genetic diversity of the batches used was not determined, it seems likely that their genetic makeup was different to each other. Thus, differences in IHHNV susceptibility found here may suggest that an intrinsic factor (i.e. genetic diversity) was the cause for such differences. These results agree with another study done with whiteleg shrimp, which indicated that the more diverse the genetic makeup of a shrimp family, the lower the susceptibility to a pathogen (Alcivar-Warren et al. 1997).

A previous work with an IHHNV-resistant selected line of *Litopenaeus stylirostris* also showed the effect of the host genetic changes in virus tolerance (Tang et al. 2000). When this “resistant” shrimp line was co-cultured with virus-naïve *Litopenaeus stylirostris*, high IHHNV-related mortalities were recorded (Tang et al. 2000). This finding showed that the virus was fully pathogenic and was carried by the “resistant” shrimp, which were rather tolerant to the virus. Such a tolerance most likely was due to a genetic change in the host and led to the following updated viral accommodation hypothesis (Flegel 2007): “crustaceans and other arthropods actively accommodate viral pathogens as persistent infections that act as a kind of memory that functions to specifically reduce the severity of disease and to dampen viral triggered apoptosis”.

In the present study, shrimp batches with the lowest IHHNV susceptibility always were IHHNV-negative by PCR. This result indicates that a persistent infection (or viral accommodation) was not the cause for reduced IHHNV susceptibility. Instead, it suggests that a genetic or physiological factor in shrimp batches may be associated to a reduced IHHNV susceptibility.

Our study is considered to be a first step to assess the value of IHHNV as a natural agent against a WSSV infection. Previous studies (Tang et al. 2003b, Bonnichon et al 2006, Melena et al. 2006) revealed that pre-infecting susceptible shrimp with IHHNV reduced infection and mortality due to WSSV. This phenomenon was first described in *Litopenaeus stylirostris* juveniles (1–3 g mean body weight [MBW]) (Tang et al. 2003b), and later it was also verified with *Litopenaeus vannamei* larvae (nauplius, zoea 1), postlarvae (pl 22), which also showed a delay in mortality (100% in controls vs. 95% in IHHNV-treated shrimp at 10 d post WSSV challenge) (Melena et al. 2006), and juveniles (3.5 g MBW) (Bonnichon et al. 2006). These studies indicate a possible role of IHHNV as a natural control agent against WSSV infection. The IHHNV causes relatively low damage to farmed shrimp and it may interfere with WSSV replication. In order to assess IHHNV as an antagonist to WSSV, features such as replication speed, pathogenesis and host susceptibility need to be known. This information will help to determine the best conditions at which IHHNV can be more effective against WSSV infection in susceptible shrimp. Shrimp susceptibility to IHHNV is therefore an important trait to consider before performing such studies. A shrimp batch highly susceptible to IHHNV may allow higher virus replication rates before a WSSV challenge. Higher amounts of IHHNV in a susceptible host may impair WSSV replication, thus inducing delayed or reduced mortalities due to a WSSV infection.

As IHHNV infection does not cause mortality to whiteleg shrimp, the present study evaluated shrimp IHHNV susceptibility by their infectivity titers using PCR. Here, the infectivity titer was a measure of virus susceptibility between shrimp batches. This method may be an indirect way to assess genetic diversity. Further studies are needed to confirm that IHHNV susceptibility may be an indicator of the reduced genetic diversity and/or endogamy of shrimp batches and/or families.

## References

[B1] Alcivar-Warren A Overstreet RM Dhar AK Astrofsky K Carr WH Sweeney J Lotz JM (1997) Genetic susceptibility of cultured shrimp (*Penaeus vannamei*) to Infectious hypodermal and hematopoietic necrosis virus and Baculovirus penaei: possible relationship with growth status and metabolic gene expression. Journal of Invertebrate Pathology 70: 190–197. doi: 10.1006/jipa.1997.4692

[B2] Bell TA Lightner DV (1987) IHHN disease of *Penaeus stylirostris*: effects of shrimp size on disease expression. Journal of Fish Diseases 10: 165–70. doi: 10.1111/j.1365-2761.1987.tb01058.x

[B3] Bonami JR Trumper B Mari J Brehelin M Lightner DV (1990) Purification and characterization of the Infectious hypodermal and haematopoietic necrosis virus of penaeid shrimps. Journal of General Virology 71: 2657–2664. doi: 10.1099/0022-1317-71-11-2657 2254754 10.1099/0022-1317-71-11-2657

[B4] Bonnichon V Lightner DV Bonami JR (2006) Viral interference between Infectious hypodermal and hematopoietic necrosis virus and white spot syndrome virus in *Litopenaeus vannamei*. Diseases of Aquatic Organisms 72: 179–184. doi: 10.3354/dao072179 17140141 10.3354/dao072179

[B5] Chayaburakul K Lightner DV Sriurairattana S Tang-Nelson K Withyachumnarnkul B (2005) Different responses to Infectious hypodermal and hematopoietic necrosis virus (IHHNV) in *Penaeus monodon* and *P. vannamei*. Diseases of Aquatic Organisms 67: 191–200. doi: 10.3354/dao067191 16408834 10.3354/dao067191

[B6] Coelho MGL Silva ACG Vila Nova CMV Neto JMO Lima ACN Feijó RG Apolinário DF Maggioni R Gesteira TCV (2009) Susceptibility of the wild southern brown shrimp (*Farfantepenaeus subtilis*) to Infectious hypodermal and hematopoietic necrosis (IHHN) and Infectious myonecrosis (IMN). Aquaculture 294: 1–4. doi: 10.1016/j.aquaculture.2009.05.023

[B7] Escobedo-Bonilla CM (2013) Application of RNA Interference (RNAi) against viral infections in shrimp: a review. Journal of Antivirals and Antiretrovirals 5: 1–12.

[B8] Escobedo-Bonilla CM Alday-Sanz V Wille M Sorgeloos P Pensaert MB Nauwynck HJ (2008) A review on the morphology, molecular characterization, morphogenesis and pathogenesis of White spot syndrome virus. Journal of Fish Diseases 31: 1–18. doi: 10.1111/j.1365-2761.2007.00877.x 18086030 10.1111/j.1365-2761.2007.00877.x

[B9] Escobedo-Bonilla CM Wille M Alday-Sanz V Sorgeloos P Pensaert MB Nauwynck HJ (2005) *In vivo* titration of White spot syndrome virus (WSSV) in SPF *Litopenaeus vannamei* by intramuscular and oral routes. Diseases of Aquatic Organisms 66: 163–170. doi: 10.3354/dao066163 16231643 10.3354/dao066163

[B10] Flegel TW (2007) Update on viral accommodation, a model for host-viral interaction in shrimp and other arthropods. Developmental and Comparative Immunology 31: 217–231. doi: 10.1016/j.dci.2006.06.009 16970989 10.1016/j.dci.2006.06.009

[B11] Kalagayan H Godin D Kanna R Hagino G Sweeney J Wyban J Brock J (1991) IHHN virus as an etiological factor in runt-deformity syndrome (RDS) of juvenile *Penaeus vannamei* cultured in Hawaii. Journal of the World Aquaculture Society 22: 235–243. doi: 10.1111/j.1749-7345.1991.tb00740.x

[B12] Krabsetsve K Cullen BR Owens L (2004) Rediscovery of the Australian strain of infectious hypodermal and haematopoietic necrosis virus. Diseases of Aquatic Organisms 61: 153–158. doi: 10.3354/dao061153 15584422 10.3354/dao061153

[B13] Lightner DV (1996) A handbook of pathology and diagnostic procedures for diseases of penaeid shrimp. World Aquaculture Society, Baton Rouge, Louisiana, USA, 349 pp.

[B14] Lightner DV (2011) Virus diseases of farmed shrimp in the Western hemisphere (the Americas): A review. Journal of Invertebrate Pathology 106: 110–130. doi: 10.1016/j.jip.2010.09.012 21215359 10.1016/j.jip.2010.09.012 PMC7172539

[B15] Lightner DV Redman RM Bell TA (1983) Infectious hypodermal and hematopoietic necrosis, a newly recognized virus disease of penaeid shrimp. Journal of Invertebrate Pathology 42: 62–70. doi: 10.1016/0022-2011(83)90202-1 6886467 10.1016/0022-2011(83)90202-1

[B16] Lotz JM (1997) Special topic review: viruses, biosecurity and specific pathogen-free stocks in shrimp aquaculture. World Journal of Microbiology and Biotechnology 13: 405–413. doi: 10.1023/A:1018572132529

[B17] Martorelli S Overstreet RM Jovonovich JA (2010) First report of viral pathogens WSSB and IHHNV in Argentine crustaceans. Bulletin of Marine Science 86: 117–131.

[B18] Melena J Bayot B Betancourt I Amano Y Panchana F Alday-Sanz V Calderón J Stern S Roch P Bonami JR (2006) Pre-exposure to Infectious hypodermal and haematopoietic necrosis virus or to inactivated white spot syndrome virus (WSSV) confers protection against WSSV in *Penaeus vannamei* (Boone) post-larvae. Journal of Fish Diseases 29: 589–600. doi: 10.1111/j.1365-2761.2006.00739.x 17026668 10.1111/j.1365-2761.2006.00739.x

[B19] Morales-Covarrubias MS Chávez-Sánchez C (1999b) Histopathological studies on wild broodstock of white shrimp *Penaeus vannamei* in the Platanitos area, adjacent to San Blas, Nayarit, Mexico. Journal of the World Aquaculture Society 30: 192–200. doi: 10.1111/j.1749-7345.1999.tb00866.x

[B20] Morales-Covarrubias MS Nunan LM Lightner DV Mota-Urbina JC Garza-Aguirre MC Chávez-Sanchez MC (1999a) Prevalence of Infectious hypodermal and hematopoietic necrosis virus (IHHNV) in wild adult blue shrimp *Penaeus stylirostris* from the northern Gulf of California, Mexico. Journal of Aquatic Animal Health 11: 296–301. doi: 10.1577/1548-8667(1999)011<0296:POIHAH>2.0.CO;2

[B21] Nunan LM Arce SM Staha RJ Lightner DV (2001) Prevalence of Infectious hypodermal and hematopoietic necrosis virus (IHHNV) and White spot syndrome virus (WSSV) in *Litopenaeus vannamei* in the Pacific Ocean off the coast of Panama. Journal of the World Aquaculture Society 32: 330–334. doi: 10.1111/j.1749-7345.2001.tb00456.x

[B22] Owens L Anderson IG Kenway M Trott L Benzie JAH (1992) Infectious hypodermal and haematopoietic necrosis virus (IHHNV) in a hybrid penaeid prawn from tropical Australia. Diseases of Aquatic Organisms 14: 219–228. doi: 10.3354/dao014219

[B23] Pantoja CR Lightner DV Holtschmit K-H (1999) Prevalence and geographic distribution of Infectious hypodermal and hematopoietic necrosis virus (IHHNV) in wild blue shrimp *Penaeus stylirostris* from the Gulf of California, Mexico. Journal of Aquatic Animal Health 11: 23–34. doi: 10.1577/1548-8667(1999)011<0023:PAGDOI>2.0.CO;2

[B24] Reed LJ Muench H (1938) A simple method of estimating 50 per cent end-points. American Journal of Hygiene 27: 493–497.

[B25] Robles-Sikisaka R Bohonak AJ McClenaghan LRJ Dhar AK (2010) Genetic signature of rapid IHHNV (Infectious hypodermal and hematopoietic necrosis virus) expansion in wild *Penaeus* shrimp populations. PLoS ONE 5: 1–11. doi: 10.1371/journal.pone.0011799 10.1371/journal.pone.0011799 PMC2909959 20668694

[B26] Shike H Dhar AK Burns JC Shimizu C Jousset FX Klimpel KR Bergoin M (2000) Infectious hypodermal and hematopoietic necrosis virus of shrimp is related to mosquito Brevidensoviruses. Virology 277: 167–177. doi: 10.1006/viro.2000.0589 11062047 10.1006/viro.2000.0589

[B27] Tang KFJ Lightner DV (2002) Low sequence variation among isolates of Infectious hypodermal and hematopoietic necrosis virus (IHHNV) originating from Hawaii and the Americas. Diseases of Aquatic Organisms 49: 93–97. doi: 10.3354/dao049093 12078987 10.3354/dao049093

[B28] Tang KFJ Lightner DV (2006) Infectious hypodermal and hematopoietic necrosis virus (IHHNV)-related sequences in the genome of the black tiger prawn *Penaeus monodon* from Africa and Australia. Virus research 118: 185–191. doi: 10.1016/j.virusres.2006.01.003 16473428 10.1016/j.virusres.2006.01.003

[B29] Tang KFJ Durand SV White BL Redman RM Mohney LL Lightner DV (2003b) Induced resistance to white spot syndrome virus infection in *Penaeus stylirostris* through pre-infection with Infectious hypodermal and hematopoietic necrosis virus – a preliminary study. Aquaculture 216: 19–29. doi: 10.1016/S0044-8486(02)00498-2

[B30] Tang KFJ Durand SV White BL Redman RM Pantoja CR Lightner DV (2000) Postlarvae and juveniles of a selected line of *Penaeus stylirostris* are resistant to Infectious hypodermal and hematopoietic necrosis virus infection. Aquaculture 190: 203–210. doi: 10.1016/S0044-8486(00)00407-5

[B31] Tang KFJ Poulos BT Wang J Redman RM Shih HH Lightner DV (2003a) Geographic variations among Infectious hypodermal and hematopoietic necrosis virus (IHHNV) isolates and characteristics of their infection. Diseases of Aquatic Organisms 53: 91–99. doi: 10.3354/dao053091 12650241 10.3354/dao053091

[B32] White DO Fenner FJ (1994) Medical Virology. Academic Press, San Diego California, 603 pp.

[B33] Yang B song XL Huang J Shi CY Liu L (2007) Evidence of existence of Infectious hypodermal and hematopoietic necrosis virus in penaeid shrimp cultured in China. Veterinary Microbiology 120: 63–70. doi: 10.1016/j.vetmic.2006.10.011 17118584 10.1016/j.vetmic.2006.10.011

