# Corrigenda: César Marcial Escobedo-Bonilla, José Luis Ibarra Rangel (2014) Susceptibility to an inoculum of infectious hypodermal and haematopoietic necrosis virus (IHHNV) in three batches of whiteleg shrimp *Litopenaeus vannamei* (Boone, 1931). ZooKeys 457: 355–365. doi: 10.3897/zookeys.457.6715

**DOI:** 10.3897/zookeys.609.9367

**Published:** 2016-08-08

**Authors:** César Marcial Escobedo-Bonilla, José Luis Ibarra Rangel

**Affiliations:** 1 Instituto Politécnico Nacional - CIIDIR Unidad Sinaloa. Blvd Juan de Dios Batiz Paredes no 250, Colonia San Joachin. Guasave, Sinaloa. Codigo Postal 81101

It came to our attention after our manuscript was published that the Institution name was missing. The full and correct institution name and address is provided here in order to acknowledge the deserved credits.

Also, authors acknowledge funding of this study by projects SIP 20110016 and SIP 20121103.

